# Developing a crisis leadership evaluation system for Chinese nursing staff during major infectious disease emergencies: a modified Delphi study

**DOI:** 10.1186/s12912-025-03050-8

**Published:** 2025-04-15

**Authors:** Changchang Chen, Xutong Zheng, Man Zhang, Luxi Zhang, Wenjie Chen, Shizhe He, Hezi Mu, Xuejun Hu, Hongjuan Lang

**Affiliations:** 1 Department of Nursing, Air Force Medical University, No. 169 Changle West Road, Xi’an, Shaanxi Province China; 2 School of Nursing, China Medical University, Shenyang, Liaoning China; 3 Intensive Care Unit, Shaanxi Provincial People’s Hospital, Xi’an, Shaanxi China; 4 Harbin Medical University, Harbin, Heilongjiang China; 5 School of Pharmacy, Yichun University, Yichun, Jiangxi China; 6 Department of Health Services, Air Force Medical University, Xi’an, Shaanxi China

**Keywords:** Nursing, Crisis leadership, Delphi, Infectious disease, Public health emergencies, Pandemic, Evaluation system

## Abstract

**Aims:**

To create a consensus on the nursing crisis leadership evaluation system during major infectious disease emergencies.

**Background:**

Crisis leadership is critical to prevent and mitigate an infectious disease infectious disease public health emergency during crisis time. However, there has been no crisis leadership evaluation system for nursing staff during major infectious diseases emergencies in China.

**Methods:**

We used a two-part modified Delphi method. Part 1 focused on creating a pool of indicators and developing an evaluation framework through a systematic literature review and a qualitative interview. Part 2 revised the indicators and built the final the evaluation system using two rounds of the Delphi surveys, following the Conducting and REporting of DElphi Studies (CREDES) guidance. Indicators were scored by a panel of experts based on the 5-point Likert scale. The weights of the indicators at each level were identified by analytical hierarchy process (AHP) methods.

**Results:**

A consensus was reached on a framework for assessing crisis leadership in nursing. Experts who met the inclusion criteria participated in round 1 (*n* = 23) and 2 (*n* = 19). The recovery rates for the two rounds of the Delphi survey were 92% and 82%. The authority coefficients (Cr) were 0.88 and 0.93, respectively, indicating the high reliability of the consultation results. The Kendall coefficients (*W*) of the two rounds were 0.106 and 0.150 (*P* < 0.001). The final consensus set comprised 6 primary indicators, 18 secondary indicators, and 38 tertiary indicators. The Weights of the six primary indicators allocated by AHP, namely loading the responsibility, heading the team, governing the situation, foreseeing the crisis, thriving on crisis, and insisting on the faith, were 0.3056, 0.2500, 0.1944, 0.1389, 0.0833, and 0.0278.

**Conclusion:**

A consensus-based, contemporary set of nursing crisis leadership evaluation systems in the context of major infectious disease emergencies has been identified. Ongoing work is needed to further develop a highly reliable scale, determine the current state of nursing crisis leadership, construct a targeted training curriculum, and implement the program into practice that managers may wish to use to assess, select, and develop the next generation of nursing leaders.

**Supplementary Information:**

The online version contains supplementary material available at 10.1186/s12912-025-03050-8.

## Background

Recent major infectious disease emergencies, such as the 2014 Ebola epidemics, the coronavirus disease 2019 (COVID-19) pandemic, and the 2022 mpox outbreak, spread rapidly worldwide and caused substantial morbidity and mortality [1]. Specifically, nearly half a million infections and over 50,000 deaths associated with novel coronavirus occurred in mainland China alone during the 2020–2022 COVID-19 period [2]. The pandemic posed a serious threat to social security, human health, and economic development. The effective control and mitigation of pandemic crises caused by infectious diseases are public health priorities.

Crises are often understood as events perceived by managers and stakeholders as unexpected, highly salient, and potentially devastating to individuals, organizations, and societies [3]. Crisis events are context-specific with varying characteristics; for example, epidemics are highly contagious and easily spread, whereas earthquakes are not. Crisis leadership refers to the process in which leaders take action to prepare for the onset of unexpected crises, respond to their salient effects, and grow from the devastating experiences of crises [4]. Crisis leadership in nursing focuses on six core attributes: clear, rapid, and honest communication; high-level collaboration; information sharing; prioritizing decision-making and equity; building trust; and competency [5]. Effective crisis leadership is critical to the survival and growth of an organization. Nursing staff, as the largest group of healthcare professionals, play an essential role in providing direct care to frontline patients and meeting the society’s health needs, especially during public health emergencies of infectious diseases. Crisis leadership is not limited to nursing managers; every nurse is a potential leader [6, 7]. We must recognize that COVID-19 will not be the last public health crisis that policymakers and healthcare professionals confront [8], and therefore nursing crisis leadership has a significant place in the domain of infectious disease prevention and control and deserves special attention.

When the COVID-19 pandemic crisis broke out, nursing leaders and their staff had to make quick decisions in unpredictable situations and promptly adjust work patterns and procedures [9]. In particular, frontline nurses faced multiple pressures: delivering life-saving treatments with limited healthcare resources, risking infection, and enduring psychological challenges such as fear, panic, and anxiety [10]. Given the multiple demands on nurses during crises, careful consideration must be given to the qualifications of nurses who comprise emergency or crisis response teams. These teams are critical frontline responders who are dispatched to provide essential medical relief and care to individuals impacted by a disaster or emergency [11].

In China, however, the nursing crisis response teams are staffed from various wards or departments. Apart from infection departments and intensive care units, nurses in general wards often lack experience in managing virulent infectious diseases and occupational protection against such diseases. Pre-service training for crisis response teams primarily relies on unannounced intensive emergency training as the main training mode, and the selection of personnel for clinical first-line emergency response teams is often based on subjective evaluation rather than systematic and objective criteria. These limitations hinder proactive and scientifically informed responses to crises. If properly managed, crisis events can serve as key turning points for positive change and offer new opportunities for development [12]. The overall level of crisis leadership among nursing staff determines, in part, the success or failure of an infectious disease crisis. A deeper understanding of nursing crisis leadership is urgently needed to prevent and control future infectious diseases emergencies and to cultivate the next generation of nurse leaders [13].

Many studies have explored crisis leadership and emphasized the need for preparedness to lead complex crisis events [14]. For example, a recent scoping review analyzed the evidence of various crisis leadership competencies during a pandemic and grouped them into task competencies (preparing and planning, communication, collaboration), people competencies (inspiring and influencing, leadership presence, empathy, and awareness), and adaptive competencies (decision making, systems thinking and sensemaking, tacit skills) [15]. Although these reviews were theory-based, describing the most common characteristics of crisis leadership [4, 5, 12, 15, 16], there was no specific research on nursing crisis leadership and the unique nature of nurses’ work during crises, as well as on the characteristics of infectious disease crisis events (e.g., COVID-19 pandemic). Notably, the literature lacks a distinct nursing crisis leadership evaluation system for major infectious disease emergencies. Additionally, several separate studies provided unsystematic conceptual frameworks of crisis leadership using subjective approaches [17, 18]; they did not follow rigorous methodologies such as PRISMA (Preferred Reporting Items for Systematic Reviews and Meta-Analyses) during development or publication [17, 18]; and many lacked theoretical underpinning [17, 18].

Therefore, we aimed to reach a Delphi-generated expert consensus on the crisis leadership characteristics of nursing staff and its evaluation indicator framework, with a focus on major infectious disease emergencies. A clear evaluation system of nursing crisis leadership is critical to minimize role confusion within nursing crisis response team and to help individuals understand their competencies in combating pandemic. In healthcare education setting, it can also inform the blueprinting of a curriculum, including supporting nursing staff, enhancing their skills through training, and strengthening their leadership to manage crises or pandemics successfully. This study provides a reference for healthcare organizations to assess, select, and nurture nursing leaders who can effectively respond to future crises.

### Theoretical framework

**Fig. 1:**
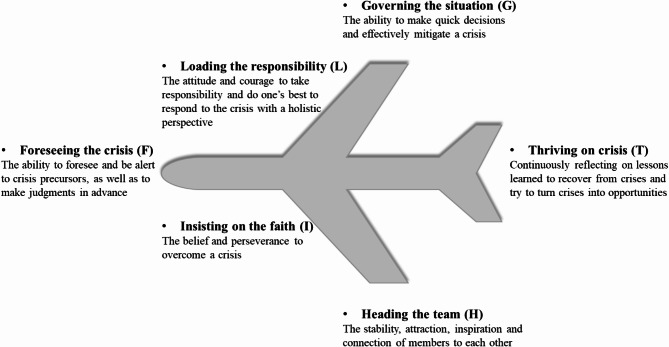
The FLIGHT model of crisis leadership

The Conceptual Model of Crisis Leadership, known as FLIGHT model, was developed by Zheng et al. based on the case of a Chinese aircraft captain [19]. Using the classical grounded theory, the FLIGHT model identified the six dimensions of crisis leadership: foreseeing the crisis (F), loading the responsibility (L), insisting on the faith (I), governing the situation (G), heading the team (H), and thriving on crisis (T). A detailed description is shown in Fig. 1. This model offers an effective framework for crisis response and mitigation from a leadership perspective, transcending the traditional stage-based analysis of crisis management (e.g., pre-crisis, mid-crisis, and post-crisis). Among the various crisis management theories, the 4R Crisis Management Theory is widely applied [20, 21]. Pioneered by Robert Heath, the 4R Crisis Management Theory posits that the three phases of a crisis (pre-crisis, mid-crisis, and post-crisis) should be managed in a continuous and dynamic cycle through the four aspects: Reduction, Readiness, Response, and Recovery, to mitigate or prevent risks [20]. Despite the different stages of crisis events, data from our preliminary research with caregivers suggest that the entire crisis process, regardless of phase, requires nursing staff to possess common traits (e.g., responsibility). Therefore, we adopted the more integrated FLIGHT Model to identify its components in the nursing field and to construct an evaluation system for nursing crisis leadership in the context of major infectious disease emergencies.

### Methods

#### Design

**Fig. 2:**
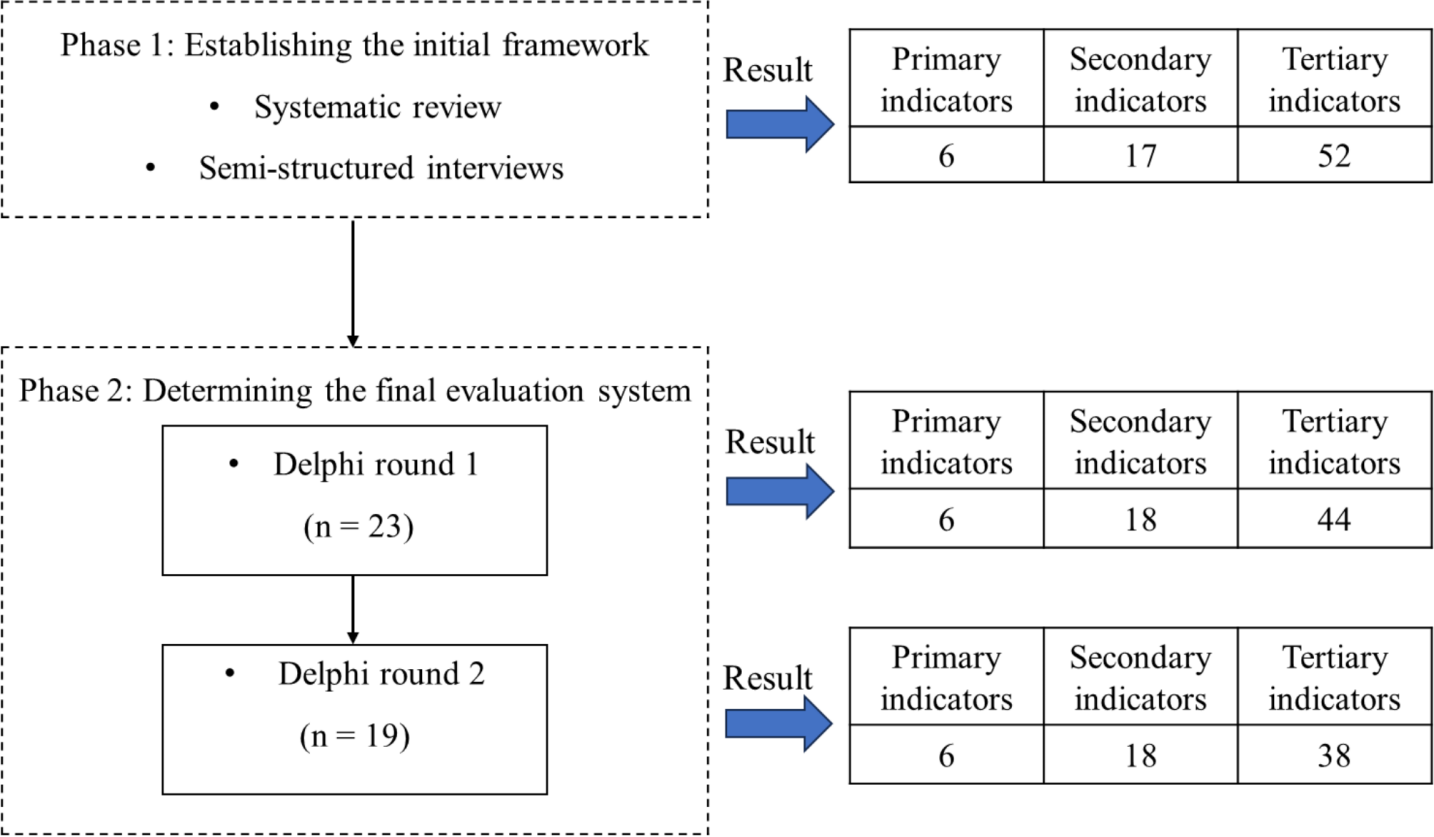
Research design flowchart

We employed the modified Delphi technique following the Conducting and REporting of DElphi Studies (CREDES) guidance, as detailed in Supplementary File 1 [22]. We conducted a two-phase design: (1) a systematic review and a qualitative interview were conducted to generate an initial set of potential indicators and develop an evaluation framework for nursing crisis leadership during major infectious disease emergencies, and (2) a two-round Delphi survey was performed to refine the indicators and finally reach a consensus on the nursing crisis leadership evaluation system in infectious public emergencies. The Delphi technique is recommended as a reliable method to achieve consensus on a research issue [23, 24]. The method involves an iterative process of repeated rounds of anonymous communication and can effectively gather the opinions of a group of experts. The first round of the classical Delphi is unstructured allowing experts to give open-ended presentations on issues they deem important [23, 24], whereas the modified Delphi method initially collects responses to a series of structured questions [25]. Studies have shown that the modified Delphi technique is more highly effective compared to the original Delphi method [25]. These steps are explained below (Fig. 2).

### Phase 1: Establishing a framework for nursing crisis leadership in major infectious disease emergencies

A systematic review of nursing crisis leadership research was performed following the PRISMA (Preferred Reporting Items for Systematic Reviews and Meta-Analyses) guidelines [26]. Chinese and English databases were comprehensively searched, including PubMed, Web of Science, China Biology Medicine (CBM), China National Knowledge Infrastructure (CNKI), China Science and Technology Journal Database (VIP), and Wanfang Database. The search was done using the search term ‘crisis leadership’ and ‘pandemic’ for articles published from the severe acute respiratory syndrome (SARS) pandemic in 2003 to February 28, 2023. Articles were included if they met the following criteria: (1) studies focused on pandemics and leadership; (2) studies were written in English or Chinese; (3) studies were published in peer-reviewed journals. Duplicates, news, and letters were excluded. Of 3560 articles identified, we screened 2998 articles by titles and abstracts to determine eligibility. Of these, 89 full-text articles were available for review. Finally, 43 articles were eligible for inclusion (see Supplementary file 2). The flowchart of the study selection is provided in Supplementary file 3. Based on the framework of the crisis leadership conceptual model, the obtained data were extracted and categorized into six dimensions: foreseeing the crisis, loading the responsibility, insisting on the faith, governing the situation, heading the team, and thriving on crisis.

In addition, we interviewed 11 nursing managers and 10 clinical nurses using semi-structured individual interviews to supplement the evaluation indicators. Detailed information about the interviewees, the interview process and the results have been published [27]. A preliminary evaluation index system was formed after iteratively review and discussion by the research team, consisting of 6 primary indicators, 17 secondary indicators, and 52 tertiary indicators.

### Phase 2: Two-Round Delphi survey to determine the final evaluation system

#### Expert panel selection

Based on the nature and size of the study, strict inclusion criteria were developed for the Delphi expert consultation to assure the scientific validity, reliability, and rigor of the evaluation system. The inclusion criteria for experts were as follows: (1) 10 years or more working experience in medical management, nursing management, or scale development; (2) a bachelor’s degree or above; (3) intermediate or higher professional title; (4) participation in COVID-19 treatment or management work; and (5) informed consent and voluntary participation in this study. Additionally, the disciplinary representation and geographical distribution of the experts were taken into account. A purposive method was used to recruit experts from different regions of China.

### Data collection

In round 1, a structured questionnaire was meticulously crafted by the research team, consisting of three parts (see Supplementary file 4). The first part is an introduction to the experts that describes the background of the study, its purpose and the requirements for completing the questionnaire. The second part is the basic information of experts, including age, working years, research direction, professional title, familiarity coefficient (Cs), and judgment coefficient (Ca) [28]. The Cs data refers to expert’s familiarity with the survey’s content, including five levels (0.20 = very unfamiliar, 0.4 = not familiar, 0.6 = generally familiar, 0.8 = familiar, 1.0 = very familiar) [28]. The Ca data, representing the experts’ judgment basis, were divided into four levels (theoretical analysis, practical work experience, domestic and foreign literature reference, and intuitive judgment) [28]. The third part is the initial nursing crisis leadership evaluation framework. The list included 52 items collected from existing literature and in-depth semi-structured interviews. Experts were requested to rate the importance of each item on a 5-point Likert scale from 1 (unimportant) to 5 (very important). A blank column was also provided for experts to give comments and additional suggestions. Prior to initiating the round 1, the questionnaire was rigorously reviewed by two experts (LHJ and HXJ) with expertise in managing and mitigating pandemics. This critical assessment aimed at testing the questionnaire’s structure and content, ascertaining its robustness for the subsequent stages.

After the first-round questionnaires were returned, the corresponding entries were modified, deleted, or added according to the feedback from the indicator screening criteria and expert comments. In round 2, experts were invited to answer the modified questionnaire in the same manner as in round 1 (see Supplementary file 5). For those statements with non-consensus, the experts provided free-text answers to newly formulated questions in the second round. Each round of the Delphi questionnaire was sent to the experts by e-mail or WeChat to avoid unintentional guiding feedback and collected within two weeks. If experts did not return within 10 days, they were reminded by email or phone. Round 1 was completed from 12/27/2022 to 01/11/2023 and round 2 from 02/22/2023 to 03/08/2023.

### Data analysis

SPSS 26.0 and Microsoft Excel were used for statistical analysis. The general information about the experts was analyzed by using frequency, rate, mean, and standard deviation (SD). The expert positive coefficient is shown by the questionnaire recovery rate, with a recovery rate > 70% is considered satisfactory [29]. Furthermore, the authority coefficients (Cr) is the degree of experts’ authority, determined by a combination of Cs (expert’s familiarity with the items) and Ca (the judgment basis). The formula is Cr = (Ca + Cs) /2. When Cr ≥ 0.70, the result is generally considered reliable [29]. The coordination degree of expert opinions is indicated by the coordination coefficient (Kendall’s W). A higher Kendall’s W value means better coordination of the items and higher consistency of expert opinions. In this study, the criteria for selecting indicators were: average importance value ≥ 3.5 points and coefficient of variation (CV) [(SD/average importance value) × 100%] ≤ 0.25, as well as combining the experts’ opinions on the modification of the items and the discussion results of the subject group to select the indicators [30]. The pre-defined cut-off for consensus on an indicator or set of similar opinions was ≥ 70% [31]. The analytical hierarchy process (AHP) is utilized to calculate the weights of the entries at each level [32].

### Ethical considerations

Participation was voluntary and the panel had the right to refuse or withdraw from the study at any time. At the beginning of the study, all participants were informed of the purpose of the study and signed an informed consent form. We obtained approval from the ethics committee of the Second Affiliated Hospital of Air Force Medical University (No. K202303-13).

## Results

### Basic information of experts

In the first round, 25 experts were invited, 23 of whom responded (response rate 92%). The 23 participants were female with a mean age of 51.04 years (SD = 7.772) and an average work experience of 30.22 years (SD = 9.327). In the second round, 19 of the 23 invited experts responded with a response rate of 82%. The mean age of the 19 respondents was 48.79 years (SD = 8.370) and the mean work experience was 27.74 years (SD = 10.082). All participants in a two-round Delphi survey were a deputy senior title or above, and a bachelor’s degree or above. The basic details are shown in Table 1.

**Table 1:** Participant demographic data

Category	Round 1, *n* (%)	Round 2, *n* (%)
Gender		
Female	23 (100.0)	19 (100.0)
Male	0 (0.0)	0 (0.0)
Age (years)		
30–39	2 (8.7)	3 (15.8)
40–49	8 (34.8)	7 (36.8)
≥ 50	13 (56.5)	9 (47.4)
Educational background		
Bachelor	13 (56.5)	10 (52.6)
Master	4 (17.4)	5 (26.3)
Doctor	6 (26.1)	4 (21.1)
Work experience (years)		
< 20	5 (21.7))	5 (26.3)
20–29	4 (17.5))	4 (21.1)
≥ 30	14 (60.8)	10 (52.6)
Title		
Senior (Professor/Senior nurse)	15 (65.2)	11 (57.9)
Associate senior (Associate professor/ Associate senior nurse)	8 (34.8)	8 (42.1)
Supervisor		
Master supervisor only	13 (56.5)	7 (36.8)
Doctoral supervisor	2 (8.7)	4 (21.1)
Not graduate supervisor	8 (34.8)	8 (42.1)
Region		
North West	9 (39.1)	6 (31.6)
North China	3 (13.0)	3 (15.8)
South West	3 (13.0)	3 (15.8)
East China	3 (13.0)	2 (10.5)
North East	3 (13.0)	3 (15.8)
Central China	2 (8.7)	2 (10.5)

### Authority and coordination of experts

In round one, the Ca was 0.9, the Cs was 0.8, and the Cr of experts was 0.88. In round two, the Ca was 1.0, the Cs was 0.9, and the Cr was 0.93. The Kendall’s W for the two rounds were 0.106 (χ^2^ = 173.041) and 0.150 (χ^2^ = 262.238), respectively, and the results were tested to be statistically significant (*P* < 0.001). The relatively low Kendall’s W values indicate a modest level of agreement among raters, suggesting that while consensus was achieved, the degree of consistency in rankings was not strong. This may be attributed to the complexity or subjectivity of the evaluated criteria. Despite the low values, the statistical significance confirms that the observed agreement is not due to chance, supporting the validity of the consensus process.

### Indicator modifications

Through a literature review and semi-structured interviews, an initial nursing crisis leadership evaluation framework was developed, including 6 primary indicators, 17 secondary indicators, and 52 tertiary indicators. After the first round, 116 comments from 14 experts were received for revisions. Consensus was reached on tier 1 indicators and a new indicator “executive ability” was added to tier 2 indicators. 35 tertiary items were deleted, added, merged, or split. Specifically, the ability to identify public health emergencies of different infectious diseases was added in the sub-dimension of event screening ability. Experts suggested that honor belongs not only to the individual, but also to the organization, so we have revised “Win honor for the individual” to “win honor for the individual and the collective”. In terms of educational guidance ability, two experts recommended to remove “provide clinical supervision to nursing students”. The reason is that during the fight against infectious diseases, where the virus spreads rapidly and patients are in critical condition, nursing students rarely participate in such situations as members of the crisis response team. It was suggested to add an item “the ability to grasp the latest evidence-based evidence” under the rapid learning ability subdimension. The second round received 52 revisions from 13 experts. The experts agreed on the first-level and second-level items and amended the content of the tertiary indicators. A high-level consensus was reached after the second Delphi survey. Finally, the nursing crisis leadership evaluation system in major infectious disease emergencies included 6 primary indicators, 18 secondary indicators, and 38 tertiary indicators (Table 2). The weights of the indicators at each tier by AHP were shown in Table 2.

**Table 2:** An evaluation system of crisis leadership among Chinese nursing staff in major infectious disease emergencies

Indicators	Mean ± SD	CV	Weight
1. Foreseeing the crisis	4.78 ± 0.60	0.126	0.1389
1.1 Information insight ability	4.83 ± 0.49	0.102	0.0494
1.1.1 Timely and rapid insight into early warning signals in the early stages of infectious disease outbreaks	4.83 ± 0.49	0.102	0.0270
1.1.2 Identify key information among the many that may confuse infectious disease events with non-infectious disease events	4.83 ± 0.49	0.102	0.0270
1.2 Event screening ability	4.83 ± 0.49	0.102	0.0710
1.2.1 Be able to initially recognize types of infectious diseases (e.g. respiratory infections, gastrointestinal infections, etc.)	4.57 ± 0.59	0.129	0.0049
1.2.2 Be able to synthesize various information to determine specific infectious disease emergencies are (e.g., COVID-19, influenza A, etc.)	4.65 ± 0.78	0.167	0.0076
1.3 Hazard predictive ability	4.91 ± 0.29	0.059	0.0494
1.3.1 Be able to predict the severe consequences of pandemics	4.78 ± 0.42	0.088	0.0194
1.3.2 Be able to assist units in developing emergency plans for infectious diseases emergencies in advance to mitigate risks	4.83 ± 0.39	0.080	0.0270
2. Loading responsibility	4.96 ± 0.21	0.042	0.3056
2.1 Big picture awareness	4.91 ± 0.29	0.059	0.0957
2.1.1 Be able to prioritize the overall interests of the organization and the general situation during critical epidemics	4.91 ± 0.29	0.059	0.0381
2.2 Responsibility	4.91 ± 0.29	0.059	0.0957
2.2.1 Be able to consciously take responsibility for the completion of nursing work, conscientious and responsible	4.91 ± 0.29	0.059	0.0317
2.2.2 Be sensitive to potential problems in the nursing process and solve them in time	4.96 ± 0.21	0.042	0.0443
2.3 Dedication	4.70 ± 0.47	0.100	0.0124
2.3.1 Be willing to sacrifice ego to fight infectious disease	4.91 ± 0.29	0.059	0.0381
3. Insisting on the faith	4.70 ± 0.56	0.119	0.0278
3.1 Sense of Mission	4.87 ± 0.34	0.071	0.0710
3.1.1 Have the determination to overcome infectious diseases and dare to rush ahead	4.78 ± 0.42	0.088	0.0194
3.1.2 Be able to lead team and foster a shared belief in overcoming outbreaks	4.91 ± 0.29	0.059	0.0381
3.2 Sense of honor	4.48 ± 0.73	0.163	0.0031
3.2.1 Have the confidence in excelling in nursing work and win honor to themselves and the collective	4.30 ± 1.02	0.237	0.0007
3.3 Willpower	4.74 ± 0.45	0.095	0.0216
3.3.1 Not giving up easily when facing numerous challenges	4.74 ± 0.86	0.182	0.0118
4. Governing the situation	4.83 ± 0.39	0.080	0.1944
4.1 Decision-making ability	4.78 ± 0.42	0.088	0.0309
4.1.1 Follow infectious disease laws, guidelines, and other relevant regulations for any decision-making in clinical work	5.00 ± 0.00	0.000	0.0499
4.1.2 Report to superiors before making decisions on complex issues	4.87 ± 0.34	0.071	0.0319
4.1.3 Be able to think critically to find the best care option before making decisions	4.78 ± 0.42	0.088	0.0194
4.1.4 Be able to conduct rapid screening of suspected cases and high-risk infections and take corresponding disposal measures quickly	5.00 ± 0.00	0.000	0.0499
4.1.5 Be able to judge the patients’ condition changes quickly and accurately in collaboration with doctors and take the best care measures in emergency situations	4.96 ± 0.21	0.042	0.0443
4.1.6 Be able to rationally evaluate the positive and negative outcomes of an important clinical decision for patients with infectious diseases	4.87 ± 0.34	0.071	0.0319
4.2 Organizational ability	4.83 ± 0.39	0.080	0.0494
4.2.1 Be able to communicate and coordinate effectively with relevant departments, patients, and families	4.91 ± 0.29	0.059	0.0381
4.2.2 Be able to manage and schedule your work time appropriately	4.91 ± 0.29	0.059	0.0381
4.3 Executive ability	4.87 ± 0.34	0.071	0.0710
4.3.1 Be able to accurately understand and quickly relay requests from senior leaders	5.00 ± 0.00	0.000	0.0499
4.3.2 Be able to do the “six things” in carrying out the work during the epidemic: norms, actions, results, changes, effectiveness, and records	4.96 ± 0.21	0.042	0.0443
4.4 Educational guidance ability	4.70 ± 0.47	0.100	0.0126
4.4.1 Be able to provide nursing-related consultation support for the prevention, treatment, and rehabilitation of infectious diseases	4.78 ± 0.42	0.088	0.0194
4.4.2 Be able to use relevant resources to offer experiential guidance to nursing peers	4.74 ± 0.54	0.114	0.0118
5. Heading the team	4.87 ± 0.34	0.071	0.2500
5.1 Empathic ability	4.83 ± 0.39	0.080	0.0494
5.1.1 Be able to put yourself in the shoes of others (leaders, peers, or patients) and think differently in nursing practice	4.58 ± 0.51	0.111	0.0194
5.2 Evocative ability	4.91 ± 0.29	0.059	0.0957
5.2.1 Stay calm and clear-headed in the face of public health emergencies of infectious diseases	4.78 ± 0.42	0.088	0.0194
5.2.2 Be able to lead by example in the fight against the epidemic	4.78 ± 0.52	0.108	0.0194
5.2.3 Be able to stimulate the internal initiation of nursing colleagues from reactive to proactive to improve the quality of infectious disease care	5.00 ± 0.00	0.000	0.0499
5.2.4 Be able to boost patients’ courage to overcome illness	4.74 ± 0.54	0.114	0.0118
6. Thriving on crisis	4.74 ± 0.45	0.095	0.0833
6.1 Reflective skills	4.91 ± 0.29	0.059	0.0957
6.1.1 Be able to reflect and review the lessons learned at all stages of the infectious disease outbreak	4.78 ± 0.42	0.088	0.0194
6.1.2 Actively participate in various forms of training activities to improve myself	4.87 ± 0.34	0.071	0.0319
6.2 Ability to grasp opportunities	4.78 ± 0.60	0.126	0.0309
6.2.1 Maintain a sense of innovation in anti-epidemic care	4.39 ± 0.94	0.214	0.0021
6.2.2 Be able to translate cross-disciplinary knowledge and artificial intelligence, etc. into infectious disease care practice	4.57 ± 0.66	0.145	0.0049
6.2.3 Be able to propose new ideas and methods to solve clinical problems during the epidemic and show innovative talents	4.57 ± 0.73	0.159	0.0049
6.3 Fast learning ability	4.91 ± 0.29	0.059	0.0957
6.3.1 Rapidly and actively grasp the etiologic features, epidemiologic characteristics, clinical features, and evidence-based evidence of the emergent infectious diseases	4.70 ± 0.47	0.100	0.0090
6.3.2 Quickly master the prevention and control of emergent infectious diseases, care systems, programs, and rescue knowledge and techniques	4.96 ± 0.21	0.042	0.0443

Note. SD: Standard deviation; CV: Coefficient of variation

## Discussion

In this study, we integrated evidence from a systematic review and semi-structured interviews, and performed a two-round modified Delphi survey to reach a consensus on nursing crisis leadership in major infectious disease emergencies. The final consensus set includes 6 primary indicators, 18 secondary indicators, and 38 tertiary indicators.

Based on the FLIGHT theoretical framework, we constructed a generic crisis leadership evaluation system for nursing staff. The framework did not distinguish between managers and clinical nurses for two reasons. First, it aligns with the WHO’s policy priority to invest in leadership development for nurses [33]. Leadership, unlike managerial competence by nature, does not rely on a formal position [34]. Second, the data obtained from the qualitative interviews confirmed this. We conducted in-depth interviews with 11 nursing managers and 10 clinical nurses, all of whom were involved in frontline crisis response during the outbreak. The results verified that the traits required for two different positional groups during an infectious disease crisis were similar. For example, managers emphasized indicators such as executive ability, responsibility and a sense of honor, which were equally indispensable for clinical nurses. The six primary abilities are critical at all stages, for example, at any stage before, during and after a crisis, when reflection and fast learning ability are required for continuous improvement. This validated the scientific validity and rationality of opting for the FLIGHT model as a framework for research, rather than by crisis phase.

Among the evaluation indicators, loading the responsibility provided the largest weight, followed by heading the team, governing the situation, foreseeing the crisis, thriving on crisis, and insisting on the faith. This suggests that loading the responsibility was the most critical indicator for the nursing crisis leadership in major infectious disease emergencies. Regarding the sub-dimensions, a sense of the big picture and responsibility are particularly prominent. The recent crisis events, such as the Ebola outbreak, have posed huge challenges to healthcare systems. Due to the inherent nature of infectious diseases (e.g., urgent time, limited information, rapid virus transmission, and life-threatening), nursing staff not only face health risks but also endure high-intensity rescue and care work. Furthermore, when confronted with major outbreaks and emergent crises, nursing staff need to adopt a holistic perspective whenever possible, take into account the entire organization, and seize critical decision-making moments to identify optimal solutions to the crisis (e.g., shortage of supplies, occupational exposure, power outage, etc.). These challenges make it particularly difficult for nursing staff, especially non-managerial nurses, to respond effectively to infectious disease crises. Therefore, hospitals should prioritize selecting nursing staff with global awareness and a strong sense of responsibility to form emergency teams, which is a key factor in effectively responding to emerging infectious disease emergencies.

Additionally, heading the team was identified as an important indicator of nursing crisis leadership in major infectious disease emergencies. It refers to the ability of nursing staff to motivate and stabilize team and collaborate effectively during major infectious diseases emergencies in this study. During the COVID-19 pandemic, numerous studies have demonstrated that frontline nursing staff experienced high levels of psychological distress, including anxiety, depression, fear, hopelessness, loss of purpose, insecurity, and posttraumatic stress disorder (PTSD) [35]. Heading the team can contribute to positive cognitive appraisal, which helps boost confidence in nursing staff to overcome the epidemic, relieve their psychological stress, and enable them to regulate emotions effectively [36]. Among the sub-dimensions of heading the team, the one with the highest weight was evocative capacity, which refers to the ability to attract, motivate, and unite people to work together. Consistent with previous research, it could inspire and evoke hope and confidence in colleagues and patients, enabling them to combat infectious diseases with a positive mindset, greater passion, and motivation. To enhance these abilities, we suggest that the key way is through effective communication, the port of emotional and psychological connection with each other. It is also vital to empathize with others, lead by example, and demonstrate confidence to build emotional trust, evoke inspiration, and stabilize the team.

Several scholars argued that the ability to control the situation was an important indicator in responding to infectious disease public health emergencies. This study found that nursing staff applied decision-making, organizational, executive, and educational guidance abilities to manage crises and respond effectively to major infectious disease emergencies. Based on feedback from the panelists, executive ability was added to the sub-dimension of control power after the first Delphi round. Previous studies pointed out that, to a certain extent, the executive ability of nursing staff is closely related to the survival and development of the hospital [37]. Executive ability in this study refers to the ability of the capacity of an individual or group to accurately and swiftly complete anti-epidemic tasks or achieve goals. Perhaps one of the greatest advantages of possessing executive ability is the speed with which actions can be implemented. Individuals with high executive competence are aware of their responsibilities and tend to propose ideas proactively and effectively, even when facing challenges, thereby enhancing the hospital’s overall crisis management. Thus, the above results highlight that executive ability is an important indicator for assessing nursing crisis leadership during major infectious disease emergencies.

The selection of experts was a critical factor influencing the quality of the Delphi method. To ensure high-quality consultation, all selected experts had frontline experience during the COVID-19 pandemic and had more than 10 years of experience in fighting viruses and managing and controlling public health emergencies of infectious diseases. We choose experts from 12 provinces, autonomous regions, municipalities in China, including Shaanxi, Shandong, Yunnan, Gansu, Xinjiang, Zhejiang, Beijing, Hubei, Hunan, Shanghai, Chongqing, and Guangdong. The effective response rates for the two rounds of expert consultation were 92% and 82% respectively, and the experts put forward a total of 168 constructive suggestions, showing the high level of engagement and support from the experts for this study. After two rounds of consultation, the authority coefficients were 0.88 and 0.93, respectively, ensuring the authority and reliability of the results. The importance scores for all items were 4.30-5.00, and CVs were 0–25%. The CV for most items decreased from round 1 to round 2, indicating that the degree of fluctuation of expert opinions was small, the degree of coordination was improved, and experts’ opinions tended to be consistent.

The variability in expert opinions observed, particularly in round 1, can be attributed to several factors. First, the diverse professional backgrounds and experiences of the experts, including nursing managers, medical managers, educators, and researchers from various regions of China, shaped their unique perspective. Second, the complexity of nursing crisis leadership in major infectious disease emergencies likely led to differing interpretations of the indicators. For instance, clinically experienced experts prioritized practical skills (e.g., executive ability), while those with administrative backgrounds emphasized strategic or emotional competencies (e.g., evocative ability, sense of honor). Finally, the iterative nature of the Delphi method, which encourages reflection and reconsideration, contributed to the variability. As experts reviewed peer feedback and reflected on their responses in round 2, their opinions evolved, leading to greater consensus. This demonstrates the Delphi method’s effectiveness in balancing diverse viewpoints while achieving consensus. Overall, this study not only provides a reasonable and scientific evaluation framework of nursing crisis leadership in major infectious disease emergencies in China but also identifies key dimensions by calculating weights of the indicators.

### Limitations

Although the Delphi technique is a robust tool and has been extensively used in various health domains, including nursing, it has certain limitations. First, the evidence was drawn from the expert consensus rather than experimental results, as the experimental or quasi-experimental designs would not be practical given real-world considerations. Second, even the CREDES standards are inconclusive about the selection of expert panels and the sample sizes needed for optimal study results [38]. While our Delphi participants represented diverse health professions and expertise, they may not fully represent the views of everyone in the industry, and further research on a broader national scope will enrich the results of this study. Additionally, the number of recruited experts for this study was relatively small but consistent with previous studies of panels of 10–50 experts [32, 39]. Third, it was difficult to achieve a higher Kendall’s *W* score because of the different backgrounds and experiences of the respondents and the fact that the meaning of the two-round questionnaires was insufficiently explained. Finally, this Delphi technique focused specifically on nursing crisis leadership in major infectious disease emergencies, with participants from China. Thus, the findings may not be generalizable to other contexts or populations. It is vital to consider the cultural and contextual differences across countries.

### Future research directions

Subsequent research on the evaluation system will focus on acceptance, translation, implementation, and evaluation. Acceptance will grow as we develop intervention strategies and operational methods to improve their operationalization. Translation, that is, translating the evaluation system of this study into a specific easy-to-use scale, will be conducted through extensive validation to refine the identified indicators of nursing crisis leadership. The validity, reliability, differentiation, and sensitivity of the assessment tools must be rigorously tested. Implementation will involve developing a targeted training curriculum, and designing and testing effective strategies to implement the program into practice. In addition, conducting quantitative studies, such as questionnaires, will help clarify the current state of crisis leadership among nursing staff at various levels. Qualitative research, such as interviews from different perspectives including hospital directors, nursing managers, nursing educators, clinical caregivers, and other stakeholders, is also recommended to provide insight into influencing factors and training needs. Finally, as scientific knowledge advances, it is imperative to constantly evaluate and incorporate the latest evidence to provide nursing staff with an optimal evaluation system and improved practices.

### Implications for practice

An evaluation system for nursing crisis leadership can strongly contribute to the leadership development of nursing staff during crisis. For nursing staff, the findings can help minimize role confusion and understand their competencies in pandemic response, thus guiding the selecting of appropriate enhancement programs to address individual deficiencies. For educators, it is necessary to identify gaps in current training programs to better prepare nurses for future pandemic crises. Existing training on infectious disease outbreaks is often incomplete, e.g., focusing on prevention, control skills, and mental health [40], yet neglecting the development of early warning skills. Additionally, trainers were selected by separate organizations without uniform and clear qualification criteria, e.g. their experience in outbreak management or handling was not reported by the studies [41]. The effectiveness of the training was difficult to assess accurately; many studies evaluated short-term outcomes after training but fail to measure long-term impacts in real-world practice [41, 42], especially regarding nurses’ ability to govern situation and thrive during crises in epidemic settings.

Our interviews revealed that frontline experience during epidemics significantly influenced nurses’ crisis leadership levels. Therefore, managers should strengthen training for new nurses to prepare future nursing leaders for infectious disease crisis. It is recommended to conduct research on emerging infectious disease outbreaks, focusing on prediction and early warning, and to develop an early warning model tailored for nursing staff. Additionally, patient care histories of individuals with typical infection symptoms should be screened and extracted from literature and medical records to create a case bank of emergency care scenarios from major infectious disease outbreaks. Virtual simulation is highly recommended due to its multiple advantages, such as providing realistic scenarios, avoiding infections caused by improper handling, and reducing site setup costs. An AI learning platform built on a nurses’ portrait system is also being suggested to intelligently assign personalized learning resources to new nurses. These measures aim to strengthen three capacities: foreseeing the crisis, governing the situation, and thriving on crisis. Additionally, managers can use diverse strategies to promote the indicators of taking the responsibility, insisting on the faith, and heading the team. Examples include mainstream media campaigns and recognition of anti-epidemic heroes. These efforts can create a united and harmonious departmental culture, enhance the attractiveness of the nursing team, and establish a role model effect.

For the global nursing community, we hope that the finding will drive reform of international nursing education to intentionally teach and develop crisis leadership for future nursing leaders. It must be noted that the current nursing education systems lack leadership training in medical schools, offer only elective courses, or fail to reach every student, which is concern [43]. We recommend integrating crisis leadership development for future pandemics into university and graduate education curricula.

At the policy level, we propose establishing and promoting a national acute infectious disease team, developing a national-level management information system to assess the team capacity, and implementing a unified management system and certification procedures. Second, we recommend developing a scientific and reasonable resource allocation plan and equipment strategy for the emergency response team. Third, a cross-regional intelligent dispatch system should be established to enhance inter-regional collaboration. At the provincial and municipal levels, specialized emergency response teams for major infectious disease outbreaks should be established in accordance with regional characteristics, operating on a ‘peacetime-crisis’ model to strengthen risk assessment and emergency preparedness for public health emergencies.

## Conclusion

Leadership has become one of the priority global strategic directions for nursing. In this study, we set out to formulate a scientifically rigorous and credible crisis leadership evaluation framework based on the Conceptual Model of Crisis Leadership. The evaluation system consists of 6 primary indicators, 18 secondary indicators, and 38 tertiary indicators. Among these, loading the responsibility, heading the team, governing the situation emerged as the top three core dimensions. The results indicated that the system can be used in the selection, training, and evaluation of nursing crisis leadership performance before, during, and after a crisis.

## Electronic supplementary material

Below is the link to the electronic supplementary material.

Supplementary file 1. CREDES checklist for reporting Delphi studies.

Supplementary file 2. The information of included studies (n = 43).

Supplementary file 3. The flowchart of the study selection.

Supplementary file 4. Modified Delphi Survey-Round 1

Supplementary file 5. Modified Delphi Survey-Round 2

## Data Availability

The data that support the findings of this study are available from the corresponding author.

## Abbreviations

AHP: Analytical hierarchy process
CBM: China Biology Medicine
CNKI: China National Knowledge Infrastructure
CREDES: Conducting and REporting of DElphi Studies
CV: Coefficient of variation
PRISMA: Preferred Reporting Items for Systematic Reviews and Meta-Analyses
SARS: Severe acute respiratory syndrome
SD: Standard deviation
VIP: China Science and Technology Journal Database
