# Supplementary figures and images for: Developing a crisis leadership evaluation system for Chinese nursing staff during major infectious disease emergencies: a modified Delphi study
Source: BMC Nurs. 2025 Apr 15;24:423. doi: 10.1186/s12912-025-03050-8 (PMC12312600; doi:10.1186/s12912-025-03050-8)

**Additional file 3. The flowchart of the study selection.**

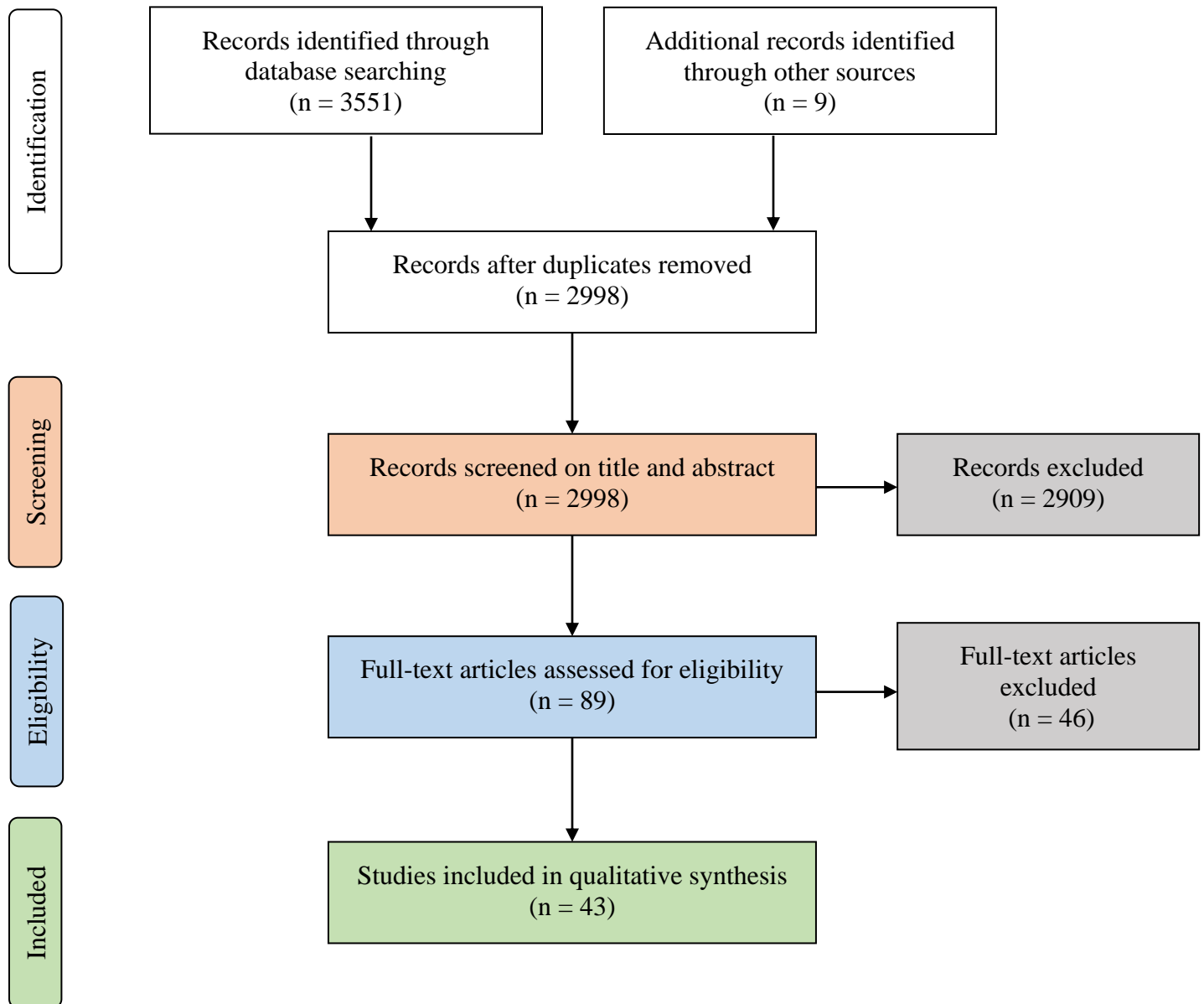

Supplement: Supplementary file 3 — The flowchart of the study selection. [file 12912_2025_3050_MOESM3_ESM.pdf]
