# Supplementary material for: Developing a crisis leadership evaluation system for Chinese nursing staff during major infectious disease emergencies: a modified Delphi study
Source: BMC Nurs. 2025 Apr 15;24:423. doi: 10.1186/s12912-025-03050-8 (PMC12312600; doi:10.1186/s12912-025-03050-8)
Supplement: Supplementary file 4 — Modified Delphi Survey-Round 1 [file 12912_2025_3050_MOESM4_ESM.docx]

**Additional file 4: Modified Delphi Study-Survey Round 1**

**Developing a crisis leadership evaluation system for Chinese nursing staff in infectious disease public health emergencies (the first round)**

Dear Expert,

We sincerely invite you to participate in the current Modified Delphi study survey (Round 1). The title of this project is "Development and Validation of the Crisis Leadership Scale for Nursing Staff in Infectious Disease Public Health Emergencies", which aims to establish a crisis leadership scale for nursing staff in infectious disease public health emergencies with good reliability and validity, and to provide a valid measurement tool for talent selection in health organizations. Based on the previous literature research, qualitative interviews and theoretical analysis, the research group has initially formed a crisis leadership evaluation system for nursing staff in infectious disease public health emergencies.

The survey consists of two sections: (a) the first part is the basic information of experts; and (b) the second part is the nursing crisis leadership evaluation framework (Table 1, 2 ,3).

*General Instructions:* The survey will take you approximately 30-90 min to complete. Your participation is voluntary, and you can withdraw from the study at any time. We promise to use the survey for academic research only and keep all data anonymous and confidential. By clicking (*yes, I consent*) below, you will indicate that you have fully read and understood the complete information in this study. Please complete the questionnaire for the first consultation round within two weeks of receipt. Your comments will be an important basis for our research! Thank you very much. For any more queries, please feel free to contact us.

On behalf of the research team, we sincerely appreciate your support and guidance!

- I hereby agree to participate and undertake this survey.
- Yes, I consent.
- No, I don’t consent.

Sincerely,

Changchang Chen

Department of Nursing, Air Force Medical University

Email: chen15137093343@163.com

Phone: XXX

27-Dec-2022

**Section A-The basic information of experts**

| 1. Age: years |
| --- |
| 2. Current position： |
| 3. Current title：  Associate senior (associate professor/associate senior nurse) Senior (professor/senior nurse) |
| 4. Please indicate the total years of experience in your field of expertise: years |
| 5. Whether you are a master or doctoral supervisor:  Not graduate supervisor Master supervisor only Doctoral supervisor |
| 6. Education level:  No degree Bachelor’s degree Master’s degree PhD |
| 7. Please indicate in which field you are an expert (choose all that applies):  Nursing management Nursing education Health emergency management  Acute and critical care Infectious disease care Clinical Medicine Other |
| 8. Have you ever been involved in the rescue or management of an outbreak of an infectious disease?  Yes No |
| 9. Your familiarity with the evaluation of nursing crisis leadership in infectious diseases public health emergencies (please select one):  Very familiar Familiar General Not familiar Very unfamiliar |
| 10. Please select your judgment basis. |

| Basis of judgement | Degree of influence | | |
| --- | --- | --- | --- |
|  | Large | General | Small |
| Theoretical analysis | √ |  |  |
| Practical work experience | √ |  |  |
| Domestic and foreign literature references | √ |  |  |
| Intuitive judgement |  |  | √ |

**Section B-****The evaluation indicators of crisis leadership among Chinese nursing staff in infectious disease public health emergencies.**

In this section of the form, statements regarding nursing crisis leadership in infectious disease are presented. Please give your rating for the importance of each indicator based on your opinion (importance level: 5 = very important, 4 = important, 3 = general, 2 = less unimportant, 1 = unimportant). Please provide your valuable suggestions in the comments column if you think the statement is not appropriate. Regarding your suggestions for additional indicators, please add them in the blank columns.

| Primary indicators | Importance level | | | | | Your revised opinions |
| --- | --- | --- | --- | --- | --- | --- |
|  | **5** | **4** | **3** | **2** | **1** |  |
| 1. Foreseeing the crisis |  |  |  |  |  |  |
| 2. Loading the responsibility |  |  |  |  |  |  |
| 3. Insisting on the faith |  |  |  |  |  |  |
| 4. Governing the situation |  |  |  |  |  |  |
| 5. Heading the team |  |  |  |  |  |  |
| 6. Thriving on crisis |  |  |  |  |  |  |
| Other additional comments | | | | | | |

**Table 1. The primary indicators questionnaire.**

**Table 2. The secondary indicators questionnaire.**

| Primary indicators | Secondary indicators | Importance level | | | | | Your revised opinions |
| --- | --- | --- | --- | --- | --- | --- | --- |
|  |  | 5 | 4 | 3 | 2 | 1 |  |
| 1. Foreseeing the crisis | 1.1 Information insight ability |  |  |  |  |  |  |
|  | 1.2 Event screening ability |  |  |  |  |  |  |
|  | 1.3 Hazard predictive ability |  |  |  |  |  |  |
| Other additional comments | | | | | | | |
| 2. Loading the responsibility | 2.1 Big picture awareness |  |  |  |  |  |  |
|  | 2.2 Responsibility |  |  |  |  |  |  |
|  | 2.3 Dedication |  |  |  |  |  |  |
| Other additional comments | | | | | | | |
| 3. Insisting on the faith | 3.1 Sense of Mission |  |  |  |  |  |  |
|  | 3.2 Sense of honor |  |  |  |  |  |  |
|  | 3.3 Willpower |  |  |  |  |  |  |
| Other additional comments | | | | | | | |
| 4. Governing the situation | 4.1 Decision-making ability |  |  |  |  |  |  |
|  | 4.2 Organizational ability |  |  |  |  |  |  |
|  | 4.3 Educational guidance ability |  |  |  |  |  |  |
| Other additional comments | | | | | | | |
| 5. Heading the team | 5.1 Empathic ability |  |  |  |  |  |  |
|  | 5.2 Evocative ability |  |  |  |  |  |  |
| Other additional comments | | | | | | | |
| 6. Thriving on crisis | 6.1 Reflective skills |  |  |  |  |  |  |
|  | 6.2 Ability to grasp opportunities |  |  |  |  |  |  |
|  | 6.3 Fast learning ability |  |  |  |  |  |  |
| Other additional comments | | | | | | | |

**Table 3. The tertiary indicators questionnaire.**

| Primary indicators | Secondary indicators | Tertiary indicators | Importance level | | | | | Your revised opinions |
| --- | --- | --- | --- | --- | --- | --- | --- | --- |
|  |  |  | 5 | 4 | 3 | 2 | 1 |  |
| 1. Foreseeing the crisis | 1.1 Information insight ability | 1.1.1 Timely and rapid insight into early warning signals in the early stages of infectious disease outbreaks |  |  |  |  |  |  |
|  |  | 1.1.2 Insight into key messages in a sea of mixed messages |  |  |  |  |  |  |
|  | 1.2 Event screening ability | 1.2.1 Be able to determine public health emergencies of infectious diseases in conjunction with multidisciplinary teams |  |  |  |  |  |  |
|  | 1.3 Hazard predictive ability | 1.3.1 Be able to predict the severe consequences of pandemics |  |  |  |  |  |  |
|  |  | 1.3.2 Be able to assist units to develop emergency plans for infectious diseases emergencies |  |  |  |  |  |  |
|  |  | 1.3.3 Be able to participate in workplace emergency drills/mock drills training with other disciplines |  |  |  |  |  |  |
|  |  | 1.3.4 Prepare mentally in advance for participation in emergency medical teams (EMTs) |  |  |  |  |  |  |
| Other additional comments | | | | | | | | |
| 2. Loading the responsibility | 2.1 Big picture awareness | 2.1.1 Be able to act in the overall interest of the organization |  |  |  |  |  |  |
|  |  | 2.1.2 Be able to consider the crisis situations comprehensively |  |  |  |  |  |  |
|  | 2.2 Responsibility | 2.2.1 Be able to consciously take responsibility for the completion of nursing work, conscientious and responsible |  |  |  |  |  |  |
|  |  | 2.2.2 Will not let personal matters interfere with the treatment of patients with infectious diseases |  |  |  |  |  |  |
|  |  | 2.2.3 Be sensitive to potential problems in the nursing process and solve them in time |  |  |  |  |  |  |
|  | 2.3 Dedication | 2.3.1 Be willing to participate in anti-epidemic missions for infectious disease public health emergencies |  |  |  |  |  |  |
|  |  | 2.3.2 Be able to coordinate time to better care for patients with infectious diseases |  |  |  |  |  |  |
| Other additional comments | | | | | | | | |
| 3. Insisting on the faith | 3.1 Sense of Mission | 3.1.1 Have the determination to overcome infectious diseases and dare to rush ahead |  |  |  |  |  |  |
|  |  | 3.1.2 Be able to lead their team or group members and have the belief to work with team members to overcome the outbreak |  |  |  |  |  |  |
|  | 3.2 Sense of honor | 3.2.1 Have the confidence to do well in the nursing work and win honor for themselves |  |  |  |  |  |  |
|  | 3.3 Willpower | 3.3.1 Not giving up easily when fighting the epidemic encounters numerous challenges |  |  |  |  |  |  |
| Other additional comments | | | | | | | | |
| 4. Governing the situation | 4.1 Decision-making ability | 4.1.1 Be able to place tubes (e.g., intravenous access, urinary catheters, etc.) quickly and effectively in critical situations |  |  |  |  |  |  |
|  |  | 4.1.2 Know the mechanism of action, dosage, and adverse effects of commonly used resuscitation drugs in the department and be able to use them correctly in critical situations |  |  |  |  |  |  |
|  |  | 4.1.3 Be able to respond to emergencies (e.g., supply shortages, patient suicides, medical malpractice, occupational exposure, power outages, fires, etc.) |  |  |  |  |  |  |
|  |  | 4.1.4 Determine principles of isolation and eradication related to infectious diseases |  |  |  |  |  |  |
|  |  | 4.1.5 Make proper judgment in using personal protective equipment (e.g., isolation gowns, goggles, etc.) for self-protection |  |  |  |  |  |  |
|  |  | 4.1.6 Select the correct emergency treatment after sharp force injuries, needlestick injuries and other related occupational exposures |  |  |  |  |  |  |
|  |  | 4.1.7 Seek advice from experts or colleagues before making decisions on complex issues |  |  |  |  |  |  |
|  |  | 4.1.8 Be able to discard inertia and think about alternative care as much as possible before making decisions |  |  |  |  |  |  |
|  |  | 4.1.9 Be able to rapidly screen suspected cases and those at high risk of infection |  |  |  |  |  |  |
|  |  | 4.1.10 Be able to judge the patients' condition changes quickly and accurately and take the best care measures in emergency situations |  |  |  |  |  |  |
|  |  | 4.1.11 Be able to rationally evaluate the positive and negative outcomes of an important clinical decision for patients with infectious diseases |  |  |  |  |  |  |
|  | 4.2 Organizational ability | 4.2.1 Be able to develop an emergency care plan based on a crisis situation |  |  |  |  |  |  |
|  |  | 4.2.2 Organize team members to carry out rescue work in an orderly manner |  |  |  |  |  |  |
|  |  | 4.2.3 Be able to resolve conflicts and contradictions in crisis situations |  |  |  |  |  |  |
|  |  | 4.2.4 Be able to communicate effectively with leaders, coworkers, patients, families, etc. |  |  |  |  |  |  |
|  |  | 4.2.5 Guide patients with infectious diseases and their families to participate in the decision-making process of disease recovery programs |  |  |  |  |  |  |
|  |  | 4.2.6 Be able to prioritize and rationalize all clinical workloads |  |  |  |  |  |  |
|  | 4.3 Educational guidance ability | 4.3.1 Be able to provide nursing-related consultation support for the prevention, treatment, and rehabilitation of infectious diseases |  |  |  |  |  |  |
|  |  | 4.3.2 Be able to use relevant resources to offer experiential guidance to nursing peers |  |  |  |  |  |  |
|  |  | 4.3.3 Be able to provide clinical supervision to nursing students |  |  |  |  |  |  |
| Other additional comments | | | | | | | | |
| 5. Heading the team | 5.1 Empathic ability | 5.1.1 Be able to put yourself in the shoes of others (leaders, peers, or patients) |  |  |  |  |  |  |
|  |  | 5.1.2 Be able to respect the views, values, and beliefs of others |  |  |  |  |  |  |
|  |  | 5.1.3 Try to meet patients' reasonable needs (e.g., patients' dietary differentiation needs, etc.) |  |  |  |  |  |  |
|  | 5.2 Evocative ability | 5.2.1 Be able to stimulate the internal initiation of nursing colleagues from reactive to proactive to improve the quality of infectious disease care |  |  |  |  |  |  |
|  |  | 5.2.2 Be able to boost patients’ courage to overcome illness |  |  |  |  |  |  |
|  |  | 5.2.3 Stay calm and clear-headed in the face of public health emergencies of infectious diseases |  |  |  |  |  |  |
|  |  | 5.2.4 Be able to lead by example in the fight against the epidemic |  |  |  |  |  |  |
| Other additional comments | | | | | | | | |
| 6. Thriving on crisis | 6.1 Reflective skills | 6.1.1 Be able to reflect and review the lessons learned at all stages of the infectious disease outbreak |  |  |  |  |  |  |
|  |  | 6.1.2 Actively participate in various forms of training activities to improve myself |  |  |  |  |  |  |
|  | 6.2 Ability to grasp opportunities | 6.2.1 Maintain a sense of innovation in anti-epidemic care |  |  |  |  |  |  |
|  |  | 6.2.2 Be able to translate cross-disciplinary knowledge and artificial intelligence, etc. into infectious disease care practice |  |  |  |  |  |  |
|  |  | 6.2.3 Be able to propose new ideas and methods to solve clinical problems during the epidemic and show innovative talents |  |  |  |  |  |  |
|  | 6.3 Fast learning ability | 6.3.1 Quickly learn ambulance techniques under the guidance of professionals |  |  |  |  |  |  |
|  |  | 6.3.2 Rapidly grasp the care, prevention and control systems and protocols for emerging and unexpected infectious diseases |  |  |  |  |  |  |
| Other additional comments | | | | | | | | |

*Thank you very much for your support! Wish everything goes well with you and happy New Year！*
