# Supplementary material for: Developing a crisis leadership evaluation system for Chinese nursing staff during major infectious disease emergencies: a modified Delphi study
Source: BMC Nurs. 2025 Apr 15;24:423. doi: 10.1186/s12912-025-03050-8 (PMC12312600; doi:10.1186/s12912-025-03050-8)
Supplement: Supplementary file 5 — Modified Delphi Survey-Round 2 [file 12912_2025_3050_MOESM5_ESM.docx]

**Additional file 5: Modified Delphi Study-Survey Round 2**

**Developing a crisis leadership evaluation system for Chinese nursing staff in infectious disease public health emergencies (the second round)**

Dear Expert,

Thank you for participating in the current Modified Delphi study survey and completing round 1. Hereby, we start Round 2 of the study. The title of this project is "Development and Validation of the Crisis Leadership Scale for Nursing Staff in Infectious Disease Public Health Emergencies", which aims to establish a crisis leadership scale for nursing staff in infectious disease public health emergencies with good reliability and validity, and to provide a valid measurement tool for talent selection in health organizations. Please help us to complete this expert consultation according to your own knowledge structure and work experience. Thank you very much. After the two rounds of consultation, we will give you a certain consultation fee.

*General Instructions:* The survey will take you approximately 30-90 min to complete. Your participation is voluntary, and you can withdraw from the study at any time. We promise to use the survey for academic research only and keep all data anonymous and confidential. By clicking (*yes, I consent*) below, you will indicate that you have fully read and understood the complete information in this study. Please complete the questionnaire for the second consultation round within two weeks of receipt. Your comments will be an important basis for our research! Thank you very much. For any more queries, please feel free to contact us.

On behalf of the research team, we sincerely appreciate your support and guidance!

- I hereby agree to participate and undertake this survey.
- Yes, I consent.
- No, I don’t consent.

Sincerely,

Changchang Chen

Department of Nursing, Air Force Medical University

Email: chen15137093343@163.com

Phone: XXX

2-Feb-2023

**The evaluation indicators of crisis leadership among Chinese nursing staff in infectious disease public health emergencies.**

In this section of the form, statements regarding nursing crisis leadership in infectious disease are presented. Please give your rating for the importance of each indicator based on your opinion (importance level: 5 = extremely important, 4 = very important, 3 = moderately important, 2 = slightly important, 1 = not important). Please provide your valuable suggestions in the comments column if you think the presentation is not appropriate. Regarding your suggestions for additional indicators, please add them in the blank columns.

| Primary indicators | Importance level | | | | | Your revised opinions |
| --- | --- | --- | --- | --- | --- | --- |
|  | **5** | **4** | **3** | **2** | **1** |  |
| 1. Foreseeing the crisis |  |  |  |  |  |  |
| 2. Loading the responsibility |  |  |  |  |  |  |
| 3. Insisting on the faith |  |  |  |  |  |  |
| 4. Governing the situation |  |  |  |  |  |  |
| 5. Heading the team |  |  |  |  |  |  |
| 6. Thriving on crisis |  |  |  |  |  |  |
| Other additional comments | | | | | | |

**Table 1. The primary indicators questionnaire.**

**Table 2. The secondary indicators questionnaire.**

| Primary indicators | Secondary indicators | Importance level | | | | | Your revised opinions |
| --- | --- | --- | --- | --- | --- | --- | --- |
|  |  | 5 | 4 | 3 | 2 | 1 |  |
| 1. Foreseeing the crisis | 1.1 Information insight ability |  |  |  |  |  |  |
|  | 1.2 Event screening ability |  |  |  |  |  |  |
|  | 1.3 Hazard predictive ability |  |  |  |  |  |  |
| Other additional comments | | | | | | | |
| 2. Loading the responsibility | 2.1 Big picture awareness |  |  |  |  |  |  |
|  | 2.2 Responsibility |  |  |  |  |  |  |
|  | 2.3 Dedication |  |  |  |  |  |  |
| Other additional comments | | | | | | | |
| 3. Insisting on the faith | 3.1 Sense of Mission |  |  |  |  |  |  |
|  | 3.2 Sense of honor |  |  |  |  |  |  |
|  | 3.3 Willpower |  |  |  |  |  |  |
| Other additional comments | | | | | | | |
| 4. Governing the situation | 4.1 Decision-making ability |  |  |  |  |  |  |
|  | 4.2 Organizational ability |  |  |  |  |  |  |
|  | 4.3 Executive ability |  |  |  |  |  |  |
|  | 4.3 Educational guidance ability |  |  |  |  |  |  |
| Other additional comments | | | | | | | |
| 5. Heading the team | 5.1 Empathic ability |  |  |  |  |  |  |
|  | 5.2 Evocative ability |  |  |  |  |  |  |
| Other additional comments | | | | | | | |
| 6. Thriving on crisis | 6.1 Reflective skills |  |  |  |  |  |  |
|  | 6.2 Ability to grasp opportunities |  |  |  |  |  |  |
|  | 6.3 Fast learning ability |  |  |  |  |  |  |
| Other additional comments | | | | | | | |

**Table 3. The tertiary indicators questionnaire.**

| Primary indicators | Secondary indicators | Tertiary indicators | Importance level | | | | | Your revised opinions |
| --- | --- | --- | --- | --- | --- | --- | --- | --- |
|  |  |  | 5 | 4 | 3 | 2 | 1 |  |
| 1. Foreseeing the crisis | 1.1 Information insight ability | 1.1.1 Timely and rapid insight into early warning signs of infectious disease outbreaks |  |  |  |  |  |  |
|  |  | 1.1.2 Identify key information among the many that may confuse infectious disease events with non-infectious disease events |  |  |  |  |  |  |
|  | 1.2 Event screening ability | 1.2.1 Be able to identify different types of public health emergencies (e.g., infectious disease category, food poisoning, etc.) |  |  |  |  |  |  |
|  |  | 1.2.2 Be able to synthesize various information to screen out which infectious disease public health emergencies are (e.g., COVID-19, influenza A, etc.) |  |  |  |  |  |  |
|  | 1.3 Hazard predictive ability | 1.3.1 Be able to predict the severe consequences of pandemics |  |  |  |  |  |  |
|  |  | 1.3.2 Be able to assist units to develop emergency plans for infectious diseases emergencies in advance to reduce hazards |  |  |  |  |  |  |
| Other additional comments | | | | | | | | |
| 2. Loading the responsibility | 2.1 Big picture awareness | 2.1.1 Be able to act in the overall interest of the organization |  |  |  |  |  |  |
|  |  | 2.1.2 Able to access information and control outbreaks from multiple sources |  |  |  |  |  |  |
|  | 2.2 Responsibility | 2.2.1 Be able to consciously take responsibility for the completion of nursing work, conscientious and responsible |  |  |  |  |  |  |
|  |  | 2.2.2 Be sensitive to potential problems in the nursing process and solve them in time |  |  |  |  |  |  |
|  | 2.3 Dedication | 2.3.1 Be willing to participate in anti-epidemic missions in infectious disease outbreaks |  |  |  |  |  |  |
|  |  | 2.3.2 Be able to coordinate time to better care for patients with infectious diseases |  |  |  |  |  |  |
| Other additional comments | | | | | | | | |
| 3. Insisting on the faith | 3.1 Sense of Mission | 3.1.1 Have the determination to overcome infectious diseases and dare to rush ahead |  |  |  |  |  |  |
|  |  | 3.1.2 Be able to lead their team or group members and have the belief to work with team members to overcome the outbreak |  |  |  |  |  |  |
|  | 3.2 Sense of honor | 3.2.1 Have confidence to do well in nursing to win honor for oneself and the collective |  |  |  |  |  |  |
|  | 3.3 Willpower | 3.3.1 Not giving up easily when facing numerous challenges |  |  |  |  |  |  |
| Other additional comments | | | | | | | | |
|  | 4.1 Decision-making ability | 4.1.1 Follow infectious disease laws, guidelines, and other relevant regulations for any decision-making in clinical work |  |  |  |  |  |  |
| 4. Governing the situation |  | 4.1.2 Report to superiors before making decisions on complex issues |  |  |  |  |  |  |
|  |  | 4.1.3 Be able to think outside the box for other care options |  |  |  |  |  |  |
|  |  | 4.1.3 Be able to rapidly screen suspected cases and those at high risk of infection |  |  |  |  |  |  |
|  |  | 4.1.4 Be able to quickly and accurately determine changes in a patient's condition and take the best nursing care possible |  |  |  |  |  |  |
|  |  | 4.1.5 Be able to rationally evaluate the positive and negative outcomes of an important clinical decision for patients with infectious diseases |  |  |  |  |  |  |
|  | 4.2 Organizational ability | 4.2.1 Be able to reasonably organize the members to complete the rescue work in an orderly manner |  |  |  |  |  |  |
|  |  | 4.2.2 Be able to resolve conflicts and contradictions in crisis situations |  |  |  |  |  |  |
|  |  | 4.2.3 Be able to guide patients with infectious diseases and their families to cooperate with nursing care |  |  |  |  |  |  |
|  |  | 4.2.4 Be able to prioritize and rationalize all clinical workloads |  |  |  |  |  |  |
|  | 4.3 Executive ability | 4.3.1 Be able to accurately understand and quickly relay requests from senior leaders |  |  |  |  |  |  |
|  |  | 4.3.2 Performs his/her own care with high quality in a limited amount of time |  |  |  |  |  |  |
|  |  | 4.3.3 Be able to work with multiple disciplines to provide innovative solutions to complex clinical problems |  |  |  |  |  |  |
|  | 4.4 Educational guidance ability | 4.4.1 Be able to provide nursing-related consultation support for the prevention, treatment, and rehabilitation of infectious diseases |  |  |  |  |  |  |
|  |  | 4.4.2 Be able to use relevant resources to offer experiential guidance to nursing peers |  |  |  |  |  |  |
| Other additional comments | | | | | | | | |
| 5. Heading the team | 5.1 Empathic ability | 5.1.1 Be able to put yourself in the shoes of others (leaders, peers, or patients) and think differently in nursing practice |  |  |  |  |  |  |
|  | 5.2 Evocative ability | 5.2.1 Stay calm and clear-headed in the face of public health emergencies of infectious diseases |  |  |  |  |  |  |
|  |  | 5.2.2 Be able to lead by example in the fight against the epidemic |  |  |  |  |  |  |
|  |  | 5.2.3 Be able to stimulate the internal initiation of nursing colleagues from reactive to proactive to improve the quality of infectious disease care |  |  |  |  |  |  |
|  |  | 5.2.4 be able to boost patients’ courage to overcome illness |  |  |  |  |  |  |
| Other additional comments | | | | | | | | |
| 6. Thriving on crisis | 6.1 Reflective skills | 6.1.1 Be able to discuss with team members regularly about tasks, methods, and efficiency of work |  |  |  |  |  |  |
|  |  | 6.1.2 Be able to reflect and review the lessons learned at all stages of the infectious disease outbreak |  |  |  |  |  |  |
|  |  | 6.1.3 Actively participate in various forms of training activities to improve myself |  |  |  |  |  |  |
|  | 6.2 Ability to grasp opportunities | 6.2.1 Maintain a sense of innovation in anti-epidemic care |  |  |  |  |  |  |
|  |  | 6.2.2 Be able to translate cross-disciplinary knowledge and artificial intelligence, etc. into infectious disease care practice |  |  |  |  |  |  |
|  |  | 6.2.3 Be able to propose new ideas and methods to solve clinical problems during the epidemic and show innovative talents |  |  |  |  |  |  |
|  | 6.3 Fast learning ability | 6.3.1 Rapidly and actively grasp the etiologic features, epidemiologic characteristics, clinical features, and evidence-based evidence of the emergent infectious diseases |  |  |  |  |  |  |
|  |  | 6.3.2 Quickly master the prevention and control of emergent infectious diseases, care systems, programs, and rescue knowledge and techniques |  |  |  |  |  |  |
| Other additional comments | | | | | | | | |

*Thank you very much for your support! Wish you a happy life!*
