# Supplementary material for: Developing a crisis leadership evaluation system for Chinese nursing staff during major infectious disease emergencies: a modified Delphi study
Source: BMC Nurs. 2025 Apr 15;24:423. doi: 10.1186/s12912-025-03050-8 (PMC12312600; doi:10.1186/s12912-025-03050-8)
Supplement: Supplementary file 6 [file 12912_2025_3050_MOESM2_ESM.odt]

Additional file 2. The information of included studies (n = 43)

| No. | Author | Year | Title | Journal | Type | Published language |
| --- | --- | --- | --- | --- | --- | --- |
| 1 | Sun et al. | 2023 | Constructing an elemental framework for public health crisis leadership - based on a crisis life cycle perspective | Party School of CPC Jiangsu Provincial Committee | Thesis | Chinese |
| 2 | Hu Z | 2023 | Crisis Leadership: Conceptual Definition and Elemental Analysis | Journal of Jiangsu Administration Institute | Article | Chinese |
| 3 | Eid et al. | 2023 | Developing local crisis leadership - A research and training agenda | Front Psychol | Review | English |
| 4 | Li et al. | 2022 | A Behavioral Approach Crisis Leadership Model Construction and Its Effectiveness | Fudan Journal (Social Sciences) | Article | Chinese |
| 5 | Bai X | 2022 | Development and Analysis of Reliability and Validity of the Model for Evaluating the Competency of Front-line Nursing Staff in A Sudden Major Infectious Disease Outbreak | Chongqing Medical University | Thesis | Chinese |
| 6 | Liu ZY | 2022 | The development and psychometric assessment of nurses’ competency questionnaire on virulent infectious diseases | China Medical University | Thesis | Chinese |
| 7 | Sha RQ | 2022 | Construction on the Index System of the Clinical Nurses' Competence Coping with Sudden Respiratory Infectious Diseases | Kunming Medical University | Thesis | Chinese |
| 8 | Dale et al. | 2022 | Crisis leadership in COVID-19: A qualitative study of Norwegian business leaders | Front Psychol | Article | English |
| 9 | Hertelendy et al. | 2022 | Crisis leadership: a case for inclusion in accredited Master of Public Health program curricula | Public Health | Review | English |
| 10 | Kvist et al. | 2022 | Nursing leadership from crisis to Postpandemic | Journal of Nursing Management | Editorial | English |
| 11 | Chekijian et al. | 2022 | Integrating crisis leadership in academic medicine | BMJ Leader | Article | English |
| 12 | Ali et al. | 2022 | Crisis, ethical leadership and moral courage: Ethical climate during  COVID-19 | Nursing Ethics | Article | English |
| 13 | Turnipseed et al. | 2022 | Crisis Leadership During and After the COVID Pandemic | the Journal of Nursing Administration | Article | English |
| 14 | Feistritzer et al. | 2022 | Complex Relational Leadership: Meeting the Challenge of Postpandemic Professional Governance | Nursing Administration Quarterly | Article | English |
| 15 | Ree et al. | 2022 | Managers' strategies in handling the COVID-19 pandemic in Norwegian nursing homes and homecare services | Leadership in Health Services | Article | English |
| 16 | Sriharan et al. | 2022 | Public Health and Health Sector Crisis Leadership During Pandemics: A Review of the Medical and Business Literature | Med Care Res Rev | Review | English |
| 17 | Bavik L et al. | 2021 | Crisis leadership: A review and future research agenda | The Leadership Quarterly | Review | English |
| 18 | Losty et al. | 2021 | Leading Through Chaos: Perspectives From Nurse Executives | Nursing Administration Quarterly | Article | English |
| 19 | Shu Q et al. | 2021 | Collaborative Leadership, Collective Action, and Community Governance against Public Health Crises under Uncertainty: A Case Study of the Quanjingwan Community in China | Int J Environ Res Public Health | Article | English |
| 20 | Kim SJ | 2021 | Crisis leadership: An evolutionary concept analysis | Applied Nursing Research: ANR | Review | English |
| 21 | Wang et al. | 2020 | Experience of comprehensive Surveillance and Response System for Acute Infectious Disease and Public Health Emergencies in Japan | Chinese Health Quality Management | Article | Chinese |
| 22 | Zheng YQ | 2020 | Crisis Leadership and Reinforcement Strategies for Leaders | Leadership Science | Article | Chinese |
| 23 | Ji et al. | 2020 | Nursing Professionalism during COVID-19 Epidemic and Its Inspiration on Talent Cultivation | Journal of Nursing (China) | Article | Chinese |
| 24 | Rohrich et al. | 2020 | Crisis Leadership | Plast Reconstr Surg | Editorial | English |
| 25 | Chuang E et al. | 2020 | "We're Not Ready, But I Don't Think You're Ever Ready." Clinician Perspectives on Implementation of Crisis Standards of Care | AJOB Empir Bioeth | Article | English |
| 26 | Geiger D et al. | 2020 | Managing enduring public health emergencies such as COVID-19: lessons from Uganda Red Cross Society’s Ebola virus disease response operation | BMJ Lead | Article | English |
| 27 | Nicola et al. | 2020 | Health policy and leadership models during the COVID-19 pandemic: A review | International Journal of Surgery | Review | English |
| 28 | Zhang CJ | 2018 | Leader's spirit of commitment and strategy to cope with the crisis | Leadership Science | Article | Chinese |
| 29 | Andrew et al. | 2018 | Ebola crisis response in the USA: Communication management and SOPs | Int J Disaster Risk Reduct | Article | English |
| 30 | Bundy et al. | 2017 | Crises and Crisis Management: Integration, Interpretation, and Research Development | Journal of Management | Article | English |
| 31 | Alkhaldi et al. | 2017 | Are you ready? Crisis leadership in a hyper-VUCA environment | American Journal of Disaster Medicine | Article | English |
| 32 | Guan Z et al. | 2016 | An Overview of Researches on Crisis Leadership: Reference and Prospect | Journal of China Executive Leadership Academy Yan’an | Article | Chinese |
| 33 | Nyenswah T | 2016 | Leadership in Times of Crisis: A Personal Reflection from the Center of the Ebola Epidemic Response in Liberia | Health Syst Reform | Article | English |
| 34 | Zhuravsky L | 2015 | Crisis leadership in an acute clinical setting: christchurch hospital, new zealand ICU experience following the february 2011 earthquake | Prehospital and Disaster Medicine | Article | English |
| 35 | Cui XM | 2014 | An Empiral Study on the Influencing Mechanism on the Crisis Leadership and Crisis Management Performance: A Stakeholder Perspctive | Fudan University | Thesis | Chinese |
| 36 | Gostin et al. | 2014 | Ebola: a crisis in global health leadership | The Lancet | Article | English |
| 37 | Deitchman et al. | 2013 | Enhancing crisis leadership in public health emergencies | Disaster Med Public Health Prep | Review | English |
| 38 | Hadley et al. | 2011 | Measuring the efficacy of leaders to assess information and make decisions in a crisis: The C-LEAD scale | The Leadership Quarterly | Article | English |
| 39 | Liu CR | 2010 | Crisis Leadership: Concepts, Elements and Models | CHINA BUSINESS & TRADE | Article | Chinese |
| 40 | Shih FJ et al. | 2009 | Surviving a life-threatening crisis: Taiwan's nurse leaders' reflections and difficulties fighting the SARS epidemic | J Clin Nurs | Article | English |
| 41 | Crouse et al. | 2008 | Crisis and emergency risk communication in a pandemic: a model for building capacity and resilience of minority communities | Health Promot Pract | Article | English |
| 42 | Austin et al. | 2007 | Pharmacy practice in times of civil crisis: The experience of SARS and the blackout in Ontario, Canada | Res Social Adm Pharm | Article | English |
| 43 | Tseng et al. | 2005 | SARS: Key factors in crisis management | The Journal of Nursing Research: JNR | Article | English |
